# Decoupling the metal insulator transition and crystal field effects of VO_2_

**DOI:** 10.1038/s41598-021-82588-4

**Published:** 2021-02-04

**Authors:** In-Hui Hwang, Chang-In Park, Sunmog Yeo, Cheng-Jun Sun, Sang-Wook Han

**Affiliations:** 1 Department of Physics Education, Institute of Fusion Science, and Institute of Science Education, Jeonbuk National University, Jeonju, 54896 Korea; 2 X-ray Science Division, Advanced Photon Source, Argonne National Laboratory, Lemont, IL 60439 USA; 3 Korea Atomic Energy Research Institute, KOMAC, Miraero 181, Gyoungju, 38180 Korea

**Keywords:** Materials science, Physics

## Abstract

VO_2_ is a highly correlated electron system which has a metal-to-insulator transition (MIT) with a dramatic change of conductivity accompanied by a first-order structural phase transition (SPT) near room temperature. The origin of the MIT is still controversial and there is ongoing debate over whether an SPT induces the MIT and whether the T_c_ can be engineered using artificial parameters. We examined the electrical and local structural properties of Cr- and Co-ion implanted VO_2_ (Cr-VO_2_ and Co-VO_2_) films using temperature-dependent resistance and X-ray absorption fine structure (XAFS) measurements at the V K edge. The temperature-dependent electrical resistance measurements of both Cr-VO_2_ and Co-VO_2_ films showed sharp MIT features. The T_c_ values of the Cr-VO_2_ and Co-VO_2_ films first decreased and then increased relative to that of pristine VO_2_ as the ion flux was increased. The pre-edge peak of the V K edge from the Cr-VO_2_ films with a Cr ion flux ≥ 10^13^ ions/cm^2^ showed no temperature-dependent behavior, implying no changes in the local density of states of V 3d *t*_2g_ and *e*_g_ orbitals during MIT. Extended XAFS (EXAFS) revealed that implanted Cr and Co ions and their tracks caused a substantial amount of structural disorder and distortion at both vanadium and oxygen sites. The resistance and XAFS measurements revealed that VO_2_ experiences a sharp MIT when the distance of V–V pairs undergoes an SPT without any transitions in either the VO_6_ octahedrons or the V 3d *t*_2g_ and *e*_g_ states. This indicates that the MIT of VO_2_ occurs with no changes of the crystal fields.

## Introduction

Since Morin reported observing the metal-to-insulator transition (MIT) of VO_2_ in 1959^1^, VO_2_ has been widely studied to understand the origin of its MIT^2–7^ and to use it in practical applications including smart windows, batteries, transistors, ultrafast switches, and gas sensors^8–15^. The MIT of VO_2_ can be induced by different factors such as heat, an electric field, doping, oxygen vacancy, photons, and a magnetic field^1,5–7,16–20^. A typical critical temperature (T_c_) of the MIT of VO_2_ is approximately 68 °C^5^. However, previous studies showed that the T_c_ is very sensitive to structural strain^21–23^. Cao and coworkers observed that VO_2_ beams with multiple domains have different T_c_ values, resulted in a dull transition^22^. Since the MIT of VO_2_ is accompanied by a first-order structural phase transition (SPT) from a monoclinic phase (M1) to a rutile phase (R) via a M2 phase, which is a mixture of the M1 and R phases, the structural changes could be related to the MIT. For the last half decade, arguments have continued as to whether this structural transition directly induces the MIT of VO_2_^2–8,24–26^. The electrical resistivity change of VO_2_ between insulator and metallic phases is approximately four orders of magnitude and the MIT is quite abrupt^5,7^. Many efforts have been made to understand the mechanism of VO_2_ MIT both theoretically and experimentally^2–8,24–29^. Many researchers attributed the abrupt MIT of VO_2_ to the SPT^2,3,24,25,30^, while others argued that the abrupt MIT can be induced by the change of carriers from holes (insulator) to electrons (metal), supporting the Mott transition of VO_2_^8,18^. The abruptness of the MIT of VO_2_ has become a further issue, in addition to its origin.

A single crystal VO_2_ has a distinct MIT temperature^22,31^ while the MIT of grained VO_2_ is dull, occurring over a wide range of temperature^7,21^. The T_c_ and the MIT curve of VO_2_ are very sensitive to structural disorder and strain^19,32–34^. When a VO_2_ film consists of grains, their structural disorder and distortion can take various forms, resulting in each grain having an individual T_c_. Furthermore, Qazilbash demonstrated that the MIT of VO_2_ could occur at slightly different temperatures for even the same grain by using infrared (IR) mapping measurements^5^. Structural disorder and defects can prevent the movement of conduction electrons and also demolish a bandgap, creating bands of impurity near the Fermi level^35^. As a result, the T_c_ of MIT can shift towards a higher or lower temperature^21–23^. Structural disorder, strain, and defects can be created by different conditions, including impurities, grain boundaries, and lattice mismatch between film and substrate^21,22,33^. For practical applications of VO_2_, the T_c_, the abruptness, and the resistance difference between metallic and insulating phases of its MIT are the most important parameters.

The T_c_ and the MIT features of VO_2_ with various impurities, including Cr, Co, W, Mo, and Ti, have been examined^17,37–44^. Added impurities in VO_2_ act as dopants, create structural disorder, and distort the atomic bond lengths^17,42,44^. Previous studies showed that the T_c_ of V_1−x_Cr_x_O_2_ shifted towards a higher temperature while it was observed at lower temperatures from V_1−x_Co_x_O_2_, V_1−x_W_x_O_2_, and V_1−x_Mo_x_O_2_ relative to that of pure VO_2_^17,37–44^. Cr^3+^ and Co^2+^ impurities in VO_2_ increased and decreased the T_c_, respectively, with little effect on the abruptness and the sharpness of the MIT curves^38,42^, although the chemical valence states of both Cr^3+^ and Co^2+^ are smaller than the 4 + of V in VO_2_. Added impurities in VO_2_ can influence the density of charge carriers in a conduction band and the structural properties and density of states. The in-situ electrical resistance and X-ray absorption fine structure (XAFS) measurements of Cr- and Co-ion implanted VO_2_ (Cr-VO_2_ and Co-VO_2_) were used to examine the contribution of local structural properties on the MIT of VO_2_. Since in a single crystal VO_2_, the correlation of the electrons (Mott insulator), the structural-driven Peierls distortion, and the crystal field effects of metal-oxide octahedrons *simultaneously* change with MIT at the same temperature, their contributions to MIT are indistinguishable. The contribution of a parameter on MIT can be distinguished from the others only when it does not occur simultaneously with the other parameters.

## Results

### The temperature-dependent electrical properties of Cr- and Co-VO_2_

**Figure 1:**
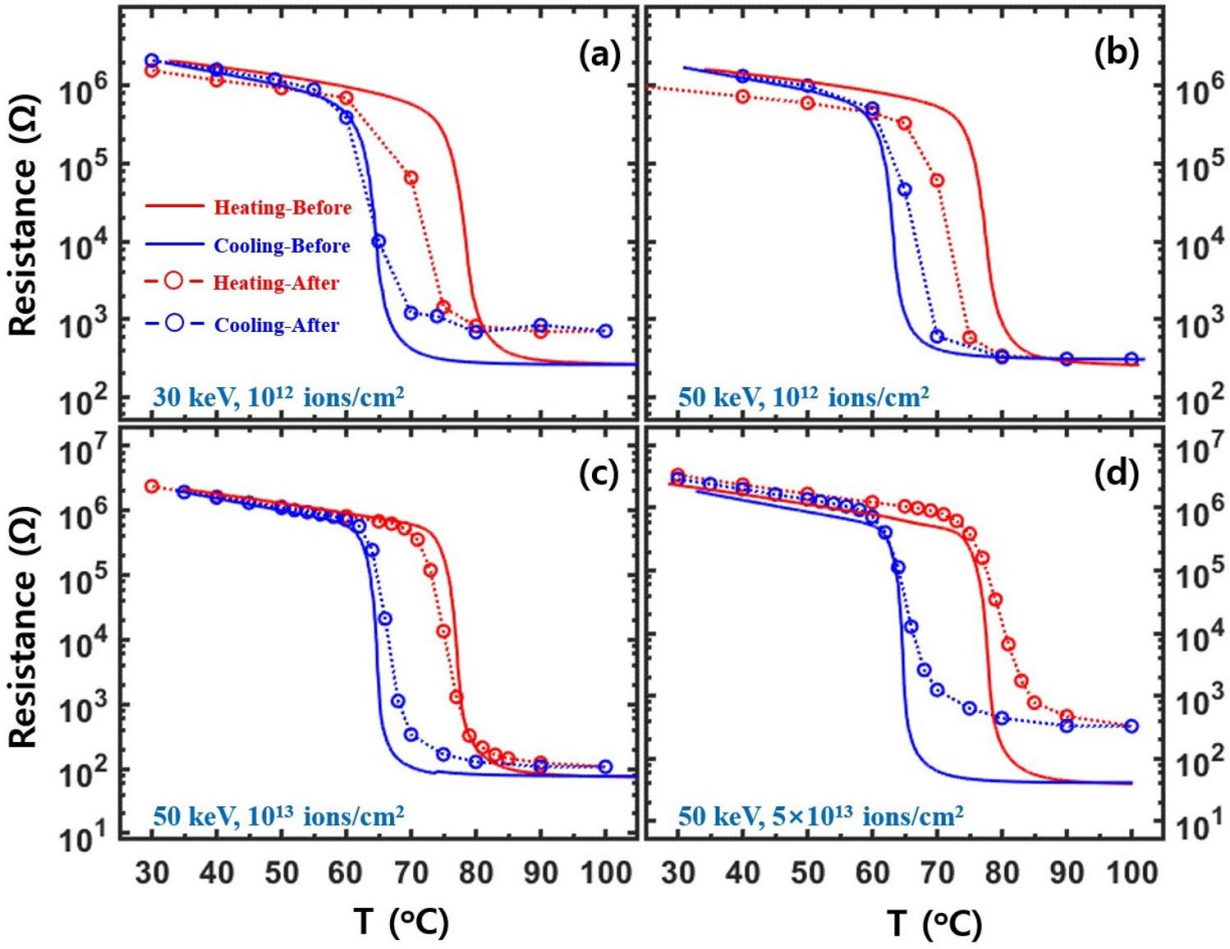
Temperature-dependent electrical resistance for Cr-VO_2_ films with a Cr ion energy and a flux of (**a**) 30 keV and 10^12^ ions/cm^2^, (**b**) 50 keV and 10^12^ ions/cm^2^, (**c**) 50 keV and 10^13^ ions/cm^2^, and (**d**) 50 keV and 5 × 10^13^ ions/cm^2^, respectively. Solid lines and circles are the resistances for the same Cr-VO_2_ film before and after Cr-ion implantation, respectively. XAFS was simultaneously measured at the temperatures of the circles. Red and blue colors indicate the resistance for heating and cooling processes, respectively. The dotted lines are a guide for the eye.

**Figure 2:**
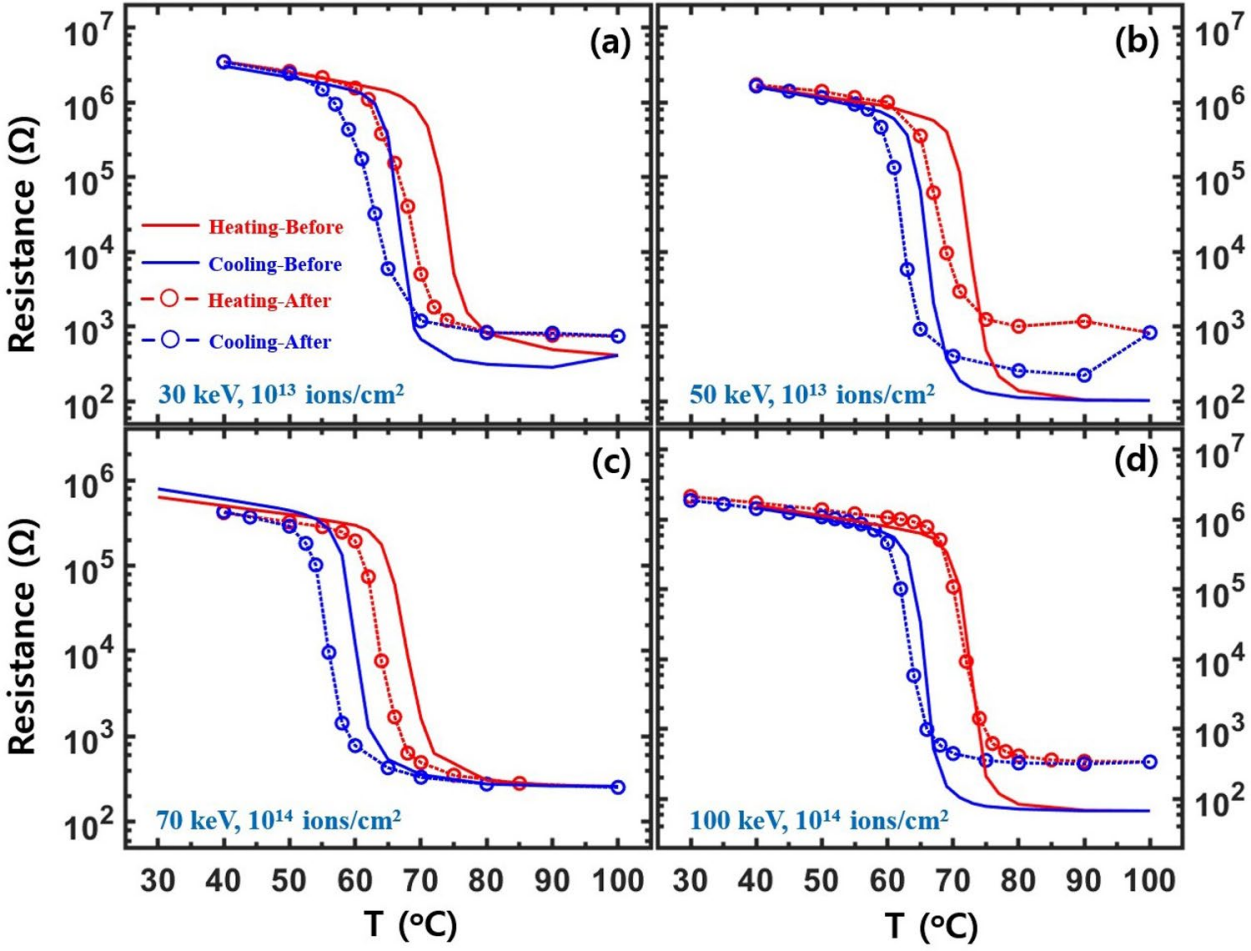
Temperature-dependent electrical resistance for Co-VO_2_ films with a Co ion energy and a flux of (**a**) 30 keV and 10^13^ ions/cm^2^, (**b**) 50 keV and 10^13^ ions/cm^2^, (**c**) 70 keV and 10^14^ ions/cm^2^, and (**d**) 100 keV and 10^14^ ions/cm^2^, respectively. Solid lines and circles are the resistances for the same Co-VO_2_ film before and after Co-ion implantation, respectively. XAFS was simultaneously measured at the temperatures of the circles. Red and blue colors indicate resistance for heating and cooling processes, respectively. The dotted lines are a guide for the eye.

Implanted Cr and Co ions can act as dopants and induce structural defects in VO_2_. Previous studies showed that the lattice constants of Cr-doped VO_2_ increased^37,39^ while the T_c_ shifted towards a higher temperature^38,39^. Figure 1 shows the temperature-dependent resistance from the Cr-VO_2_ films before and after Cr-ions implantation. The typical T_c_ value of single crystal VO_2_ is approximately 68 °C^5,31^. However, the T_c_ value of a VO_2_ film is substantially affected by structural strain and disorder and varies according to the substrate^21,32,32^. The T_c_ of ~78 °C for the pristine VO_2_ films in Fig. 1 before the ion implantation is ascribed to structural strain due to a lattice mismatch between the VO_2_ films and Al_2_O_3_ substrates^21,33,34^. The T_c_ values of pristine VO_2_ films in Fig. 2, particularly in Fig. 2(c), are somewhat lower than that in Fig. 1. The T_c_ values suggest that the growth conditions of VO_2_ films in Fig. 1 were somewhat different from those in Fig. 2, although the difference was not conscious during growth. A small deviation of the characteristics of the pristine VO_2_ films does not seriously affect the main conclusions of this study because the changes of MIT features before and after ion implantation are directly compared from the same specimen. The resistance curves of the Cr-VO_2_ films with a flux of 10^12^ ions/cm^2^ show that the T_c_ values during heating and cooling shift towards lower and higher temperatures relative to those before Cr ion implantation, respectively. As a result, the width of a hysteresis loop from the Cr-VO_2_ films, particularly with a low energy of Cr ions, is significantly narrower than that of the pristine VO_2_. For Cr ion fluxes of 10^13^ ions/cm^2^ and 5 × 10^13^ ions/cm^2^, the resistance curves become similar to that of the pristine VO_2_. At a flux of 5 × 10^13^ ions/cm^2^, the T_c_ value is ~ 2 degrees higher than that before ion implantation, as shown in Fig. 1(d). This is consistent with the previous studies of V_1−x_Cr_x_O in which the T_c_ was shifted towards a higher temperature^38,39^. The T_c_ increase of 2 degrees of Cr-VO_2_ roughly corresponds to the Cr concentration of  ~ 3%, compared to a previous study of V_1−x_Cr_x_O_2_^39^. The T_c_ shift and the resistance changes of Cr-VO_2_ can be understood in terms of structural disorder and doping effects due to implanted Cr ions. The structural damage due to implanted ions is discussed in detail in the supplementary materials.

Zou and coworkers reported that the width of the hysteresis loop of temperature-dependent resistance for VO_2_ was reduced by Cr doping and that the MIT features of VO_2_ disappeared at a Cr concentration ratio of 14%^39^. The resistance measurements of the Cr- and Co-VO_2_ films show that the MIT characteristics of VO_2_ mostly disappear when the flux of Cr and Co ions exceeds 10^15^ ions/cm^2^, which corresponds to ~ 50% of the VO_2_ cells being hit by implanted ions. The probability of a conventional cell of a film being hit by implanted ions can be estimated as $P\left(x\right)=1-A{\int }_{0}^{x}F\left(x\right)dx$, where *F*(x) is the distribution function of ions in a film and *A* is the normalization factor. The details of the probability are described in the supplementary materials. The ion tracks and the local disorder due to implanted ions likely destroy the MIT characteristics. The T_c_ shift of the Cr-VO_2_ films with an ion flux of 10^12^ ions/cm^2^ suggests that the structural disorder due to ion implantation dominantly contributes to the electrical properties because of the negligible doping effect at a concentration ratio of 0.00023%. The concentration ratio is discussed in the supplementary materials in detail. When the ion flux increases over 10^13^ ions/cm^2^, the doping effects due to the implanted ions may influence the MIT of VO_2_ because of the extremely low charge carrier density of insulating VO_2_, although structural disorder still dominantly affects the MIT.

Figure 2 shows the temperature-dependent electrical resistance of the Co-VO_2_ films with different fluxes and energies of Co ions. The distribution of implanted Co ions in VO_2_ is quite similar to that of Cr ions, as shown in the supplementary materials. When Co ions with a flux of 10^13^ ions/cm^2^ and an energy of ≤ 70 keV are implanted on VO_2_ films, the T_c_ values for both heating and cooling processes shift towards lower temperatures relative to those before the ion implantation. This is consistent with Co-doped VO_2_^42^. The width reduction of the resistance hysteresis loop is similar to that of the Cr-VO_2_ films with low ion fluxes. The resistance curves of the Co-VO_2_ films with an energy of 30–50 keV and a flux of 10^14^ ions/cm^2^ show very weak MIT features near the T_c_ of 65 °C (data not shown here). When the energy of Co ions is increased over 70 keV with a flux of 10^14^ ions/cm^2^, the Co-VO_2_ films show sharp MIT features, as shown in Fig. 2(c, d). SRIM calculations^45^ showed that the Co ions with an energy of 30 keV can become concentrated near the surface of a single crystal VO_2_. Since our VO_2_ films consist of grains with a mean size of ~ 170 nm^36^, implanted ions with the energy of 30–50 keV can affect the entire film through the grain boundaries and the lateral surfaces of the grains^46^. A lack of MIT features in Co-VO_2_ with a Co ion energy of 30 keV and a flux of 10^14^ ions/cm^2^ (data not shown here) can be ascribed to a substantial structural disorder and distortion existing in the entire film due to the ion implantation. Structural disorder and distortion in Cr- and Co-VO_2_ films which are created due to the implanted ions may not be uniformly distributed and can be more concentrated on near the surface than the bottom because the ion energy of several tens keV is insufficient to create a uniform defect in VO_2_ films with a mean thickness of ~ 130 nm.

At a Co ion energy of 100 keV, the resistance curves of the Co-VO_2_ films show sharp MIT features and the T_c_ values become similar to those before implantation during both heating and cooling. This is substantially different from those for a low energy of Co ions and sharply contrasts to previous works of Co-added VO_2_^42^. The ion-flux-dependent behavior of T_c_ values of Co-VO_2_ is similar to that of Cr-VO_2_, as shown in Fig. 1. As the flux of both Co and Cr ions exceeds a certain value, T_c_ shifts towards a higher and lower temperature relative to that for a low flux during heating and cooling, respectively, while the sharpness of MIT is not greatly affected, as shown in Figs. 1 and 2. A similar behavior of T_c_ was also observed from Ti-added VO_2_^44^. When the flux of Cr and Co ions with the energy of 30–50 keV is larger than 10^14^ ions/cm^2^, the MIT features are significantly diminished. The critical flux of the ions increases when ion energy increases, as shown in Fig. 2. This is an evidence that ions with a lower energy more effectively create structural disorder, particularly near the surface, than the ions with a higher energy.

### The temperature-dependent XANES and the pre-edge peaks of Cr-VO_2_ and Co-VO_2_

**Figure 3:**
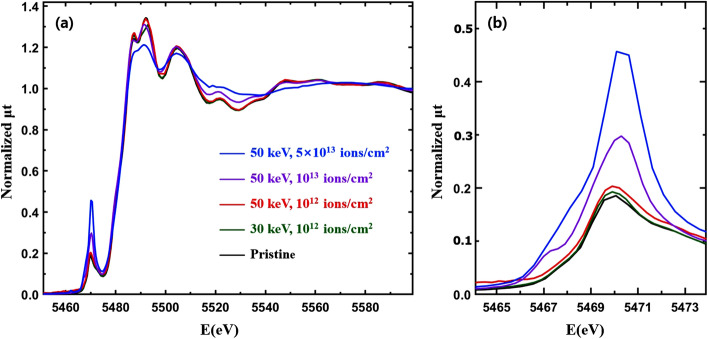
(**a**) Normalized total X-ray absorption (μt) for Cr-VO_2_ films with different Cr energies and fluxes at the V K edge as a function of the incident X-ray energy at the room temperature. The films are the same as those used for the resistance measurements, as shown in Fig. 1, and (**b**) is a magnified image of the pre-edge peaks in (**a**).

Implanted Cr^3+^ and Co^2+^ ions can affect the charge currier density of conduction bands and the local density of states around the V atoms in VO_2_. X-ray absorption near edge structure (XANES) detects the local density of empty states around a probing atom^7^. Figure 3 shows XANES from the Cr-VO_2_ films at the V K edge. The main absorption edge energy near 5478 eV from the Cr-VO_2_ films is nearly identical to that of a pristine VO_2_ film, which indicates that the chemical valance state and the 4p states of the V atoms in VO_2_ are little affected by implanted Cr ions at a flux ≤ 5 × 10^13^ ions/cm^2^. The intensity of the pre-edge peak near 5470 eV from the Cr-VO_2_ films increases dramatically for an ion flux ≥ 10^13^ ions/cm^2^, but only increases slightly for a flux of 10^12^ ions/cm^2^ relative to that of the pristine VO_2_. The pre-edge peaks consist of two peaks corresponding to the *t*_2g_ and *e*_g_ states of V 3d orbitals of VO_2_, which are separated by approximately 2.0 eV^7^. The intensity increase of the pre-edge peak at the V K edge was also observed from Ti-added VO_2_^44^. Since a lack of doping effects is expected when Ti^4+^s are replaced at V^4+^ sites in VO_2_, the pre-edge peak changes are mainly attributed to structural changes around the V atoms. The position of the pre-edge peak is shifted by approximately 0.5 eV for a Cr ion flux ≥ 10^13^ ions/cm^2^ relative to that of the pristine VO_2_, as shown in Fig. 3 (b). These intensity increases and the position shift of the pre-edge peak might be due to local structural distortion around the V atoms.

**Figure 4:**
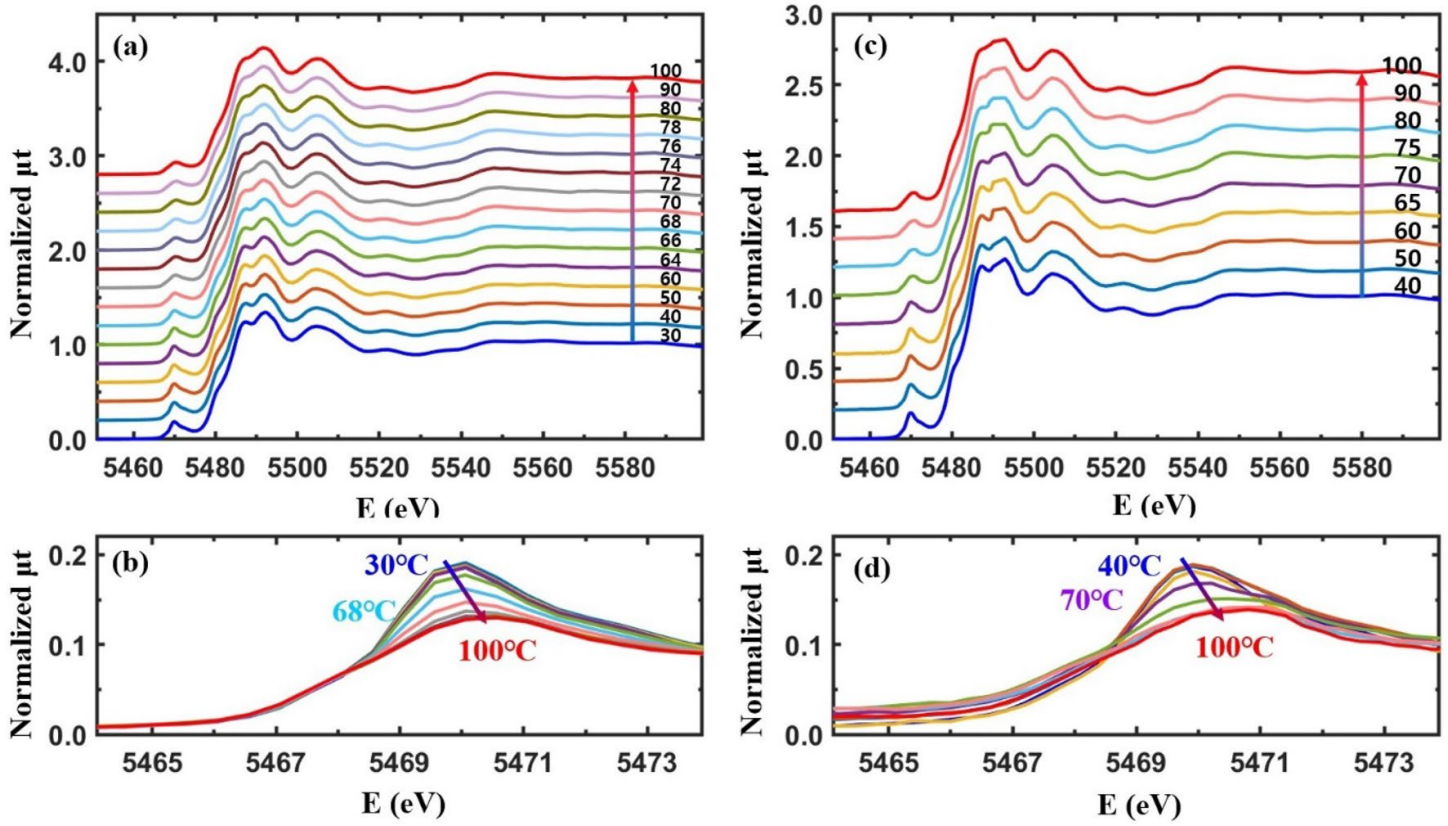
(**a**, **c**) Normalized temperature-dependent XANES (μt) from a pristine VO_2_ film and the Cr-VO_2_ film with a Cr ion energy of 30 keV and a flux of 10^12^ ions/cm^2^ during heating. (**b**, **d**) are the magnified images of the pre-edge peaks in (**a**, **c**), respectively. Data in (**a**, **c**) are vertically shifted for clarity.

Figure 4(a, b) show the temperature-dependent XANES and pre-edge peaks, respectively, from a pristine VO_2_ film in the temperature range of 30–100 °C. The main absorption edge is almost unaffected by the increasing temperature while the pre-edge shows a temperature-dependent behavior. In a pristine VO_2_ film, the dull pre-edge peak corresponds to the *t*_2g_ and *e*_g_ bands which have the energy difference of ~ 2.0 eV^7^. The direct band gap of VO_2_ is ~ 0.65 eV at room temperature^47,48^ and the Fermi level lies in the lower *t*_2g_ band^2,3,7^. Figure 3(d) shows that the intensity of the pre-edge peak decreases and the position shifts towards a higher energy when VO_2_ is heated from 30 to 100 °C. The separation of the two pre-edge peaks does not change greatly but the peak positions shift by ~ 0.5 eV towards a higher energy when the structural symmetry of VO_2_ changes from M1 to a rutile (or M2) phase^7^. The pre-edge peak of the pristine VO_2_ shows a shift at ~ 68 °C, as shown in Fig. 4(b). This is prior to the T_c_ of ~ 75 °C. The temperature-dependent behavior of the pre-edge peak is directly related to the local structural changes around the V atoms^7^.

**Figure 5:**
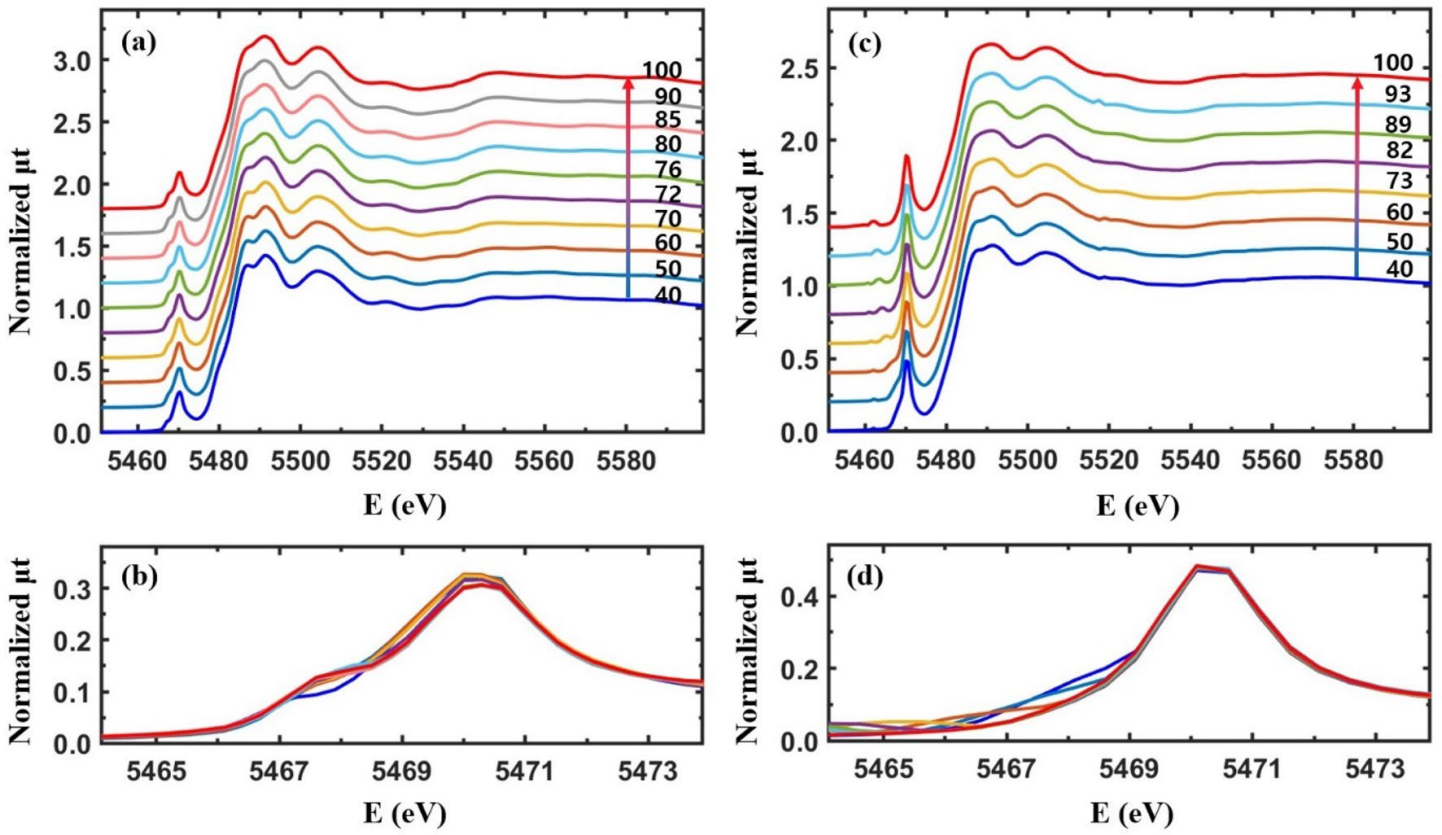
(**a**, **c**) Temperature-dependent XANES from Cr-VO_2_ films with a Cr energy of 50 keV and fluxes of 10^13^ ions/cm^2^ and 5 × 10^13^ ions/cm^2^, respectively. (**b**, **d**) are the magnified images of the pre-edge peaks in (**a**, **c**), respectively. Data in (**a**, **c**) are vertically shifted for clarity.

Electrical resistance measurements from Cr-VO_2_ films with even a low Cr ion flux show substantial changes in the T_c_ value of the MIT relative to that before ion implantation, as shown in Fig. 1. XANES and the pre-edge peak reveal that a Cr-VO_2_ film with a small flux of Cr ions shows similar behavior as that of pristine VO_2_, as shown in Fig. 4(c, d). The pre-edge peak from the Cr-VO_2_ film shifts at ~ 68 °C and ~ 65 °C while the T_c_ values of MIT are ~ 71 °C and ~ 65 °C during heating and cooling, respectively, as shown in Fig. 1(a). The pre-edge peak transitions roughly agree with the MITs of the film, although they do not occur simultaneously at the same temperature. This is consistent with pristine VO_2_^7^. For a Cr ion flux ≥ 10^13^ ions/cm^2^, the intensity of the pre-edge dramatically increases relative to that of pristine VO_2_ while the resistance curves are comparable to that before ion implantation. Figure 5(a, c) show the temperature-dependent XANES of the Cr-VO_2_ films with a flux ≥ 10^13^ ions/cm^2^. The pre-edge peaks of the films show nearly no temperature dependence in the temperature range of 40–100 °C. This sharply contrasts to that of pristine VO_2_ and Cr-VO_2_ with a low flux of the Cr ion beam. The resistance curves of the Cr-VO_2_ films show clear MIT features, as shown in Fig. 1(c, d). This temperature independence of the pre-edge peaks of the Cr-VO_2_ films is obvious evidence confirming that the pre-edge peak at the V K edge is irrelevant to the MIT of VO_2_. The pre-edge peak of a single crystal VO_2_ accidently changes with temperature due to the transition of local structural properties and the local density of states around the V atoms. This study suggests that a structural disorder can remove the correlation between the pre-edge peak and the electrical properties of VO_2_.

**Figure 6:**
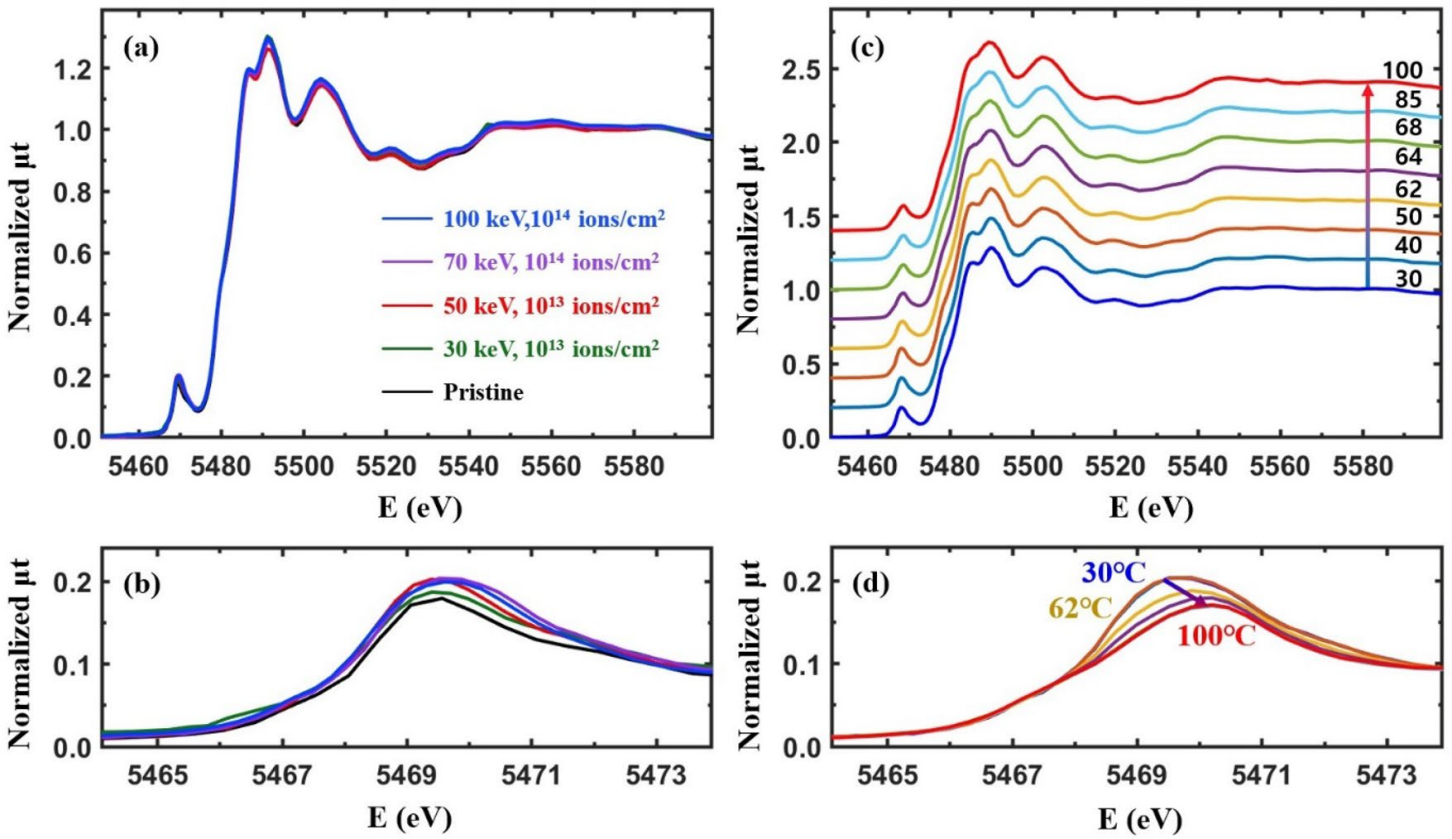
(**a**) Normalized total X-ray absorption (μt) for Co-VO_2_ films with different Co ion energies and fluxes at the V K edge as a function of the incident X-ray energy at room temperature. The films are the same as those in Fig. 2. (**c**) Temperature-dependent XANES for Co-VO_2_ with a Co energy of 70 keV and a flux of 10^14^ ions/cm^2^. (**b**, **d**) are the magnified images of the pre-edge peaks in (**a**, **c**), respectively. Data in (**c**) are vertically shifted for clarity.

Figure 6(a) shows XANES at the V K edge from Co-VO_2_ films with different energies and Co ion fluxes. The main absorption edges of the Co-VO_2_ films are nearly identical to that of pristine VO_2_. This confirms the permanence of both the chemical valance states and the local density of states around the V atoms in the Co-VO_2_ films, relative to those of the pristine VO_2_ film. The intensity of the pre-edge peaks slightly increases at a Co ion flux of 10^14^ ions/cm^2^. The XANES of Co-VO_2_ films is quite different from that of Cr-VO_2_ films, as shown in Fig. 3. Figure 6(c) shows the temperature-dependent XANES of Co-VO_2_ with a Co ion energy of 70 keV and a flux of 10^14^ ions/cm^2^. No significant changes in the main absorption edge energy of the Co-VO_2_ film are observed in the temperature range of 30–100 °C. The pre-edge peak behavior of Co-VO_2_ is similar to that of the pristine VO_2_ but it is completely different from that of Cr-VO_2_ with a Cr flux ≥ 10^13^ ions/cm^2^. The pre-edge peak shifts at 62 °C towards a higher energy, as shown in Fig. 6(d), which is slightly prior to the T_c_ of 65 °C, as shown in Fig. 2(c). The different behaviors of the pre-edge peaks from the Cr-VO_2_ and Co-VO_2_ films are mainly attributed to the different local structural properties around the V atoms.

### Local structural properties around vanadium atoms of Cr-VO_2_ and Co-VO_2_

**Figure 7:**
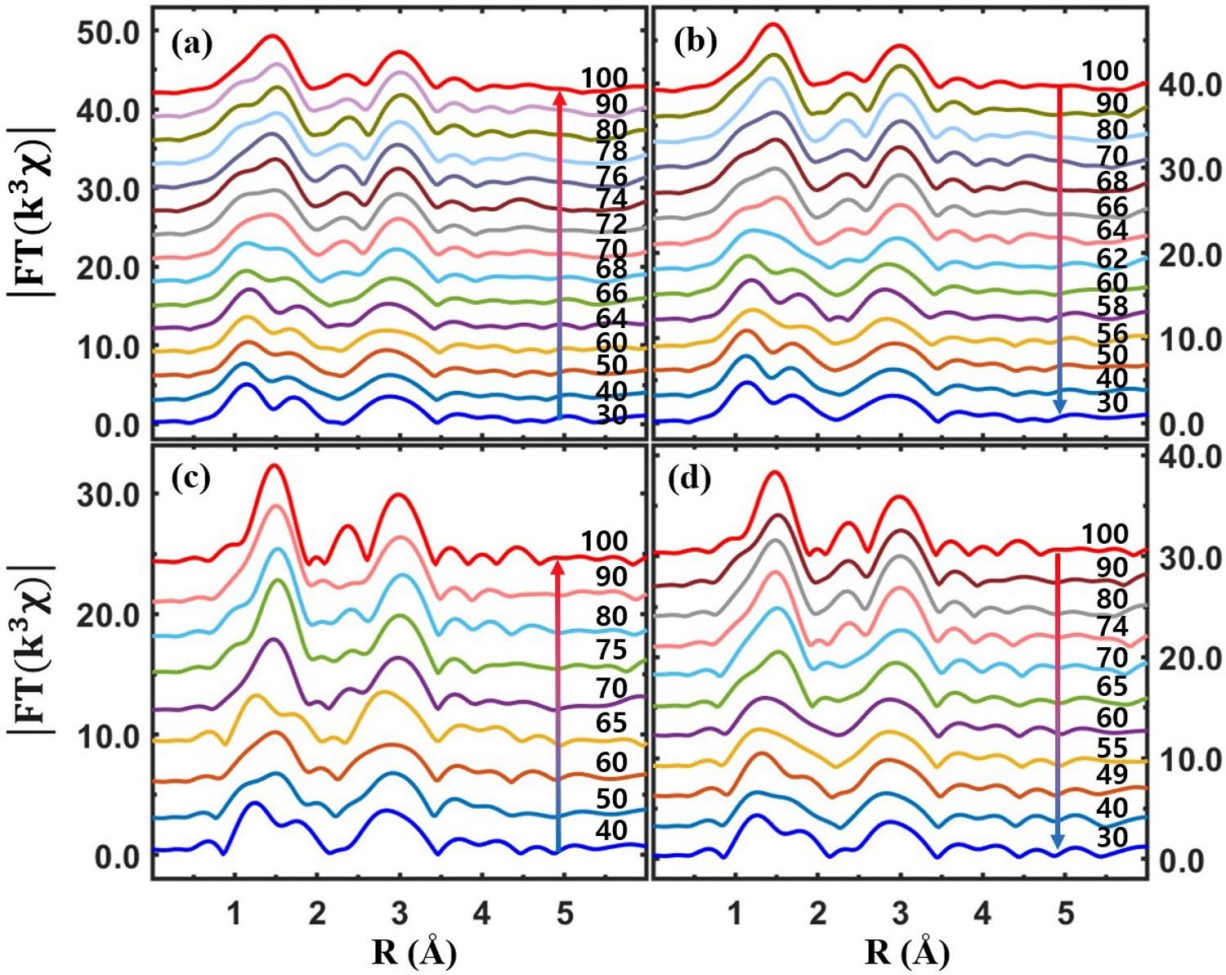
Magnitude of Fourier transformed EXAFS (|FT(k^3^χ)|) as a function of the distance from a V atom at different temperatures. (**a**, **b**) EXAFS from pristine VO_2_ for heating and cooling, respectively. (**c**, **d**) EXAFS from Cr-VO_2_ with a Cr ion energy of 50 keV and a flux of 10^12^ ions/cm^2^ for heating and cooling, respectively. The data are vertically shifted for clarity.

Extended XAFS (EXAFS), which is small oscillations above the main absorption edge, as partially shown in Figs. 3, 4, 5 and 6, can detect the local structural properties around a probing atom^50–52^. After the atomic background was determined using the IFEFFIT software package^53^, EXAFS was obtained and analyzed using the standard procedure^54^. Raw EXAFS in *k*-space is presented in the supplementary materials. Figure 7 shows Fourier transformed EXAFS from the pristine VO_2_ and Cr-VO_2_ films in *r*-space. The peak positions of EXAFS correspond to the mean atomic distances from a V atom. They are approximately 0.3 Å shorter than the true atomic positions because the phase shift of back-scattered photoelectrons is not accounted for. Figure 7(a, b) show temperature-dependent EXAFS for the pristine VO_2_ film for heating and cooling, respectively. The two peaks near 1.5 Å correspond to the six V–O pairs of a VO_6_ octahedron in VO_2_ at lower temperatures; these become one sharp peak at higher temperatures because VO_2_ has monoclinic and rutile (or M2) phases at low and high temperatures, respectively. A peak near 3.0 Å, mainly corresponding to eight vertex V atoms, slightly moves towards a longer distance in the rutile (or M2) phase, compared to that in M1. The EXAFS data of a pristine VO_2_ were quantitatively fitted to the EXAFS theory^51^ and the fit results are described elsewhere in literatures^7,36^. The SPTs of the pristine VO_2_ film are observed at ~ 70 °C and ~ 62 °C for heating and cooling, respectively. The SPT temperatures do not match with either the T_c_ of MIT or the pre-edge peak transitions. This agrees with previous reports^7,33^. From the Cr-VO_2_ film with a Cr ion energy of 50 keV and a flux of 10^12^ ions/cm^2^, the SPTs are observed at 68 °C and 63 °C for heating and cooling, respectively, as shown in Fig. 7(c, d). The temperature difference between the SPTs for heating and cooling is approximately 5 °C, which is comparable to the resistance curve after ion-implantation, as shown in Fig. 1(a). This strongly suggests that the electrical property changes and the T_c_ shifts of Cr-VO_2_ films are highly related to the structural changes due to ion implantation. In addition, this indicates that the implanted Cr ions with the energy of 50 keV cause structural disorder and distortion around V atoms in the entire Cr-VO_2_ film.

**Figure 8:**
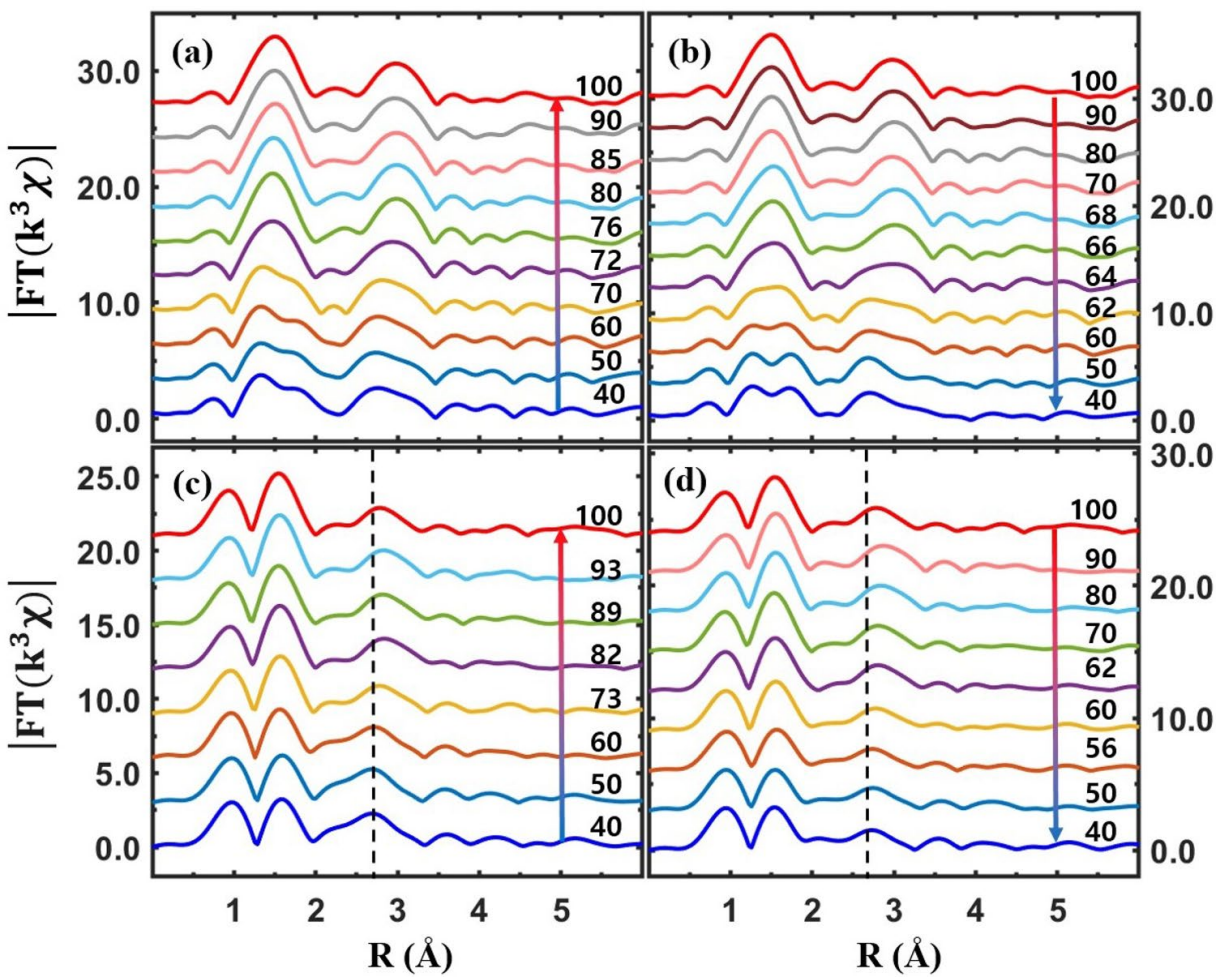
Magnitude of Fourier transformed EXAFS (|FT(k^3^χ)|) as a function of the distance from a V atom at different temperatures. (**a**, **b**) EXAFS of Cr-VO_2_ with a Cr ion energy of 50 keV and a flux of 10^13^ ions/cm^2^ for heating and cooling, respectively. (**c**, **d**) EXAFS of Cr-VO_2_ with a Cr ion energy of 50 keV and a flux of 5 × 10^13^ ions/cm^2^ for heating and cooling, respectively. The data are vertically shifted for clarity. The vertical dashed lines indicate the position of eight V atoms located at the vertexes of a rutile VO_2_.

An obvious MIT is observed from the Cr-VO_2_ films with a flux of Cr ions ≥ 10^13^ ions/cm^2^ with no changes of the pre-edge peaks in the temperature range of 30–100 °C. Figure 8 shows EXAFS from the Cr-VO_2_ films with a Cr ion flux ≥ 10^13^ ions/cm^2^. At this Cr ion flux, there are clear SPTs near 72 °C and 64 °C for heating and cooling, respectively, as shown in Fig. 8(a, b). The SPT temperatures are comparable to the MIT T_c_ values of 75 °C and 65 °C for heating and cooling, respectively, as shown in Fig. 1(c). This result is substantially different from that of a pristine VO_2_, which shows very different the T_c_ values of MIT and SPT during both heating and cooling. The similar T_c_ values of the MIT and the SPT in Cr-VO_2_ and Co-VO_2_ may suggest that Cr- and Co-VO_2_ films structurally soften relative to a pristine VO_2_ film due to ion implantation. Figure 8(c, d) show Fourier-transformed EXAFS of Cr-VO_2_ with a flux of 5 × 10^13^ ions/cm^2^. EXAFS shows no SPT of the first two peaks in 1.0–2.0 Å in the temperature range of 40–100 °C, indicating no SPT of the VO_6_ octahedron in Cr-VO_2_. There are slight changes in the position and the shape of the EXAFS peaks in the *r*-range of 2.2–3.3 Å, as shown in Fig. 8(c, d), which correspond to ten V atoms: two V atoms are located above and below along the *b*-axis and the other eight are located at the vertexes of the rutile VO_2_. The position and the shape of the V peak near 3.0 Å are evidently changed at the T_c_ of the SPT of a pristine VO_2_. A small shift of the V peak of the Cr-VO_2_ film with a flux of 5 × 10^13^ ions/cm^2^ occurs at 73 °C and 61 °C for heating and cooling, respectively. The small shift of the V peak is reproducible and consistent during heating and cooling processes, although it is quite weak. The T_c_ values of sharp MITs from the Cr-VO_2_ film are observed at 80 °C and 65 °C for heating and cooling, respectively, as shown in Fig. 1(d). No transitions in either the pre-edge peak or in the V–O distance of the Cr-VO_2_ film with a flux of 5 × 10^13^ ions/cm^2^ are observed, whereas a small shift occurs in the V–V distance. This indicates that both the pre-edge peak and the VO_6_ octahedron, which are directly related to the crystal field effects, are nearly irrelevant to the MIT of VO_2_. The structural change of the V sites could drive the MIT of VO_2_, although the V sites cannot maintain even a correct rutile symmetry above the T_c_ due to structural disorder. The transitions of the pre-edge peaks and the VO_6_ octahedrons occur accidentally with the SPT of VO_2_ crystals because the pre-edge peak is very sensitive to the nearest neighboring atoms around a probing atom and the V–O distance changes with the SPT. The EXAFS of the Cr-VO_2_ films suggests that a structural change of the V sites is related to the MIT, although the temperatures of the structural changes are not identical to the T_c_ values of the MIT during both heating and cooling.

**Figure 9:**
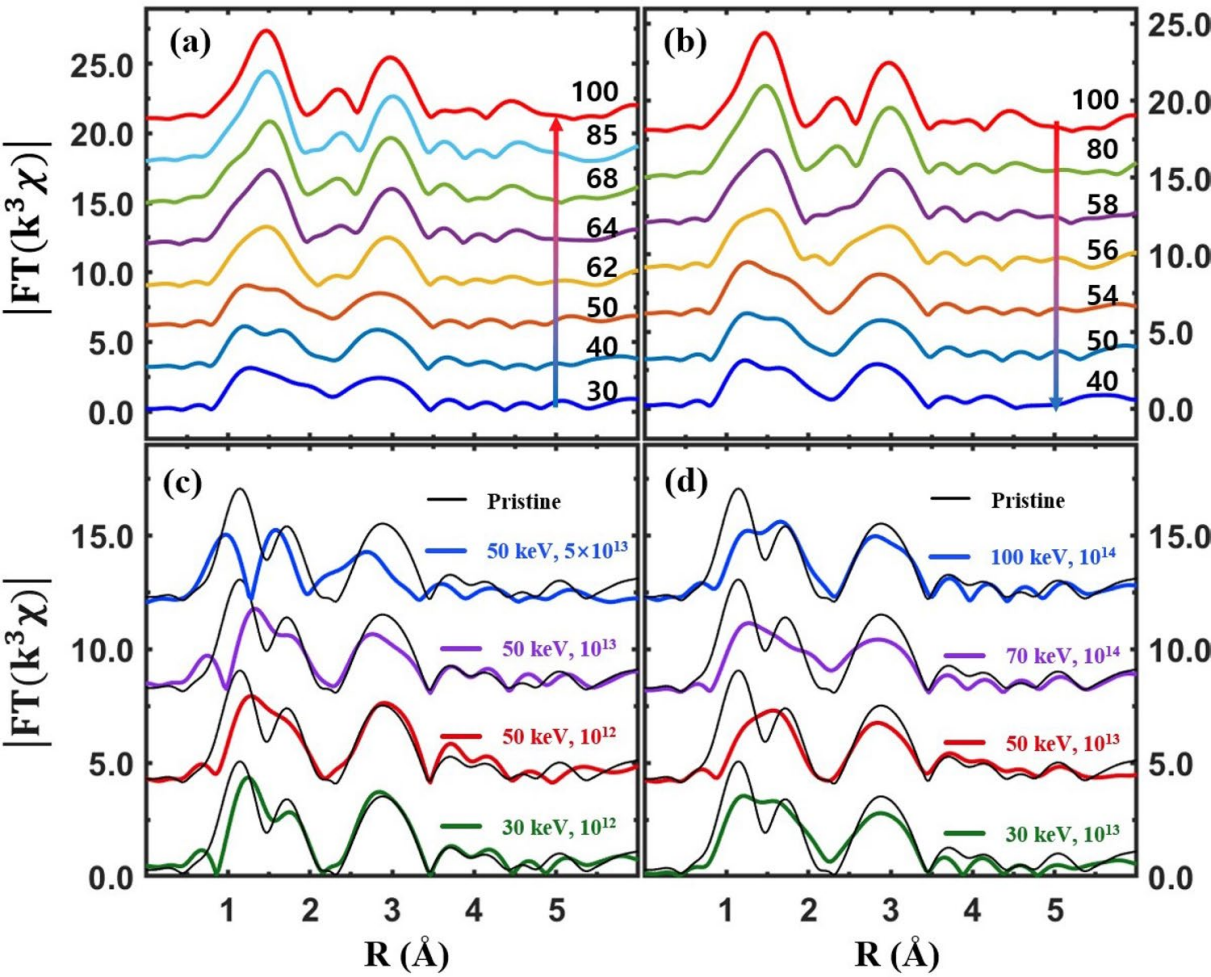
(**a**, **b**) EXAFS (|FT(k^3^χ)|) from Co-VO_2_ with a Co ion energy of 70 keV and a flux of 10^14^ ions/cm^2^ for heating and cooling, respectively. (**c**, **d**) EXAFS of Cr-VO_2_ and Co-VO_2_ films with different energies and fluxes, respectively, at room temperature. The data are vertically shifted for clarity.

Figure 9(a, b) show temperature-dependent EXAFS from Co-VO_2_ with a Co ion flux of 10^14^ ions/cm^2^. EXAFS shows the SPTs of the films occurring at ~ 62 °C and ~ 56 °C for heating and cooling, respectively, which are comparable to the T_c_ values of 65 °C and 55 °C, as shown in Fig. 2(c). The SPT of Co-VO_2_ simultaneously appears at both O and V atomic sites at the same temperature. The SPT of Co-VO_2_ is similar to that of pristine VO_2_ but it is quite different from that of Cr-VO_2_ with a Cr ion flux of 5 × 10^13^ ions/cm^2^. The distortion of atomic pairs in Cr-VO_2_ and Co-VO_2_ is more obviously seen when the EXAFS data are directly compared to those of pristine VO_2_, as shown in Fig. 9(c, d). The EXAFS of pristine VO_2_ shows two obvious peaks in the *r*-space of 1.0–2.0 Å, which correspond to six V–O pairs. When the ion flux increases, the EXAFS peak intensity is decreased, the shape is deformed, and the positions are shifted relative to those of the pristine VO_2_. The significant deformation of the first two peaks of the Cr-and Co-VO_2_ films indicates that the VO_6_ octahedrons are seriously distorted due to the implanted ions. When the flux of Cr ions is larger than 5 × 10^13^ ions/cm^2^, VO_2_ cannot maintain standard VO_6_ octahedrons. EXAFS reveals that for an ion energy of 50 keV and a flux of 10^13^ ions/cm^2^, the first peaks of Co-VO_2_ are more distorted than that of Cr-VO_2_. The second peak of EXAFS at ~ 3.0 Å, which mainly corresponds to eight V atoms at the vertexes of a rutile phase VO_2_, is also affected by the implanted ions but the distortion of the V–V pairs is less significant than that of the V–O pairs, as shown in Fig. 9(c, d). For a Cr ion flux of 10^12^ ions/cm^2^, the intensity and the shape of the second peak from Cr-VO_2_ with an energy of both 30 keV and 50 keV are similar to those of the pristine VO_2_, implying that the V sites are slightly affected by the implanted Cr ions. As the ion flux increases, the structural distortion of the V sites also increases in both Cr-VO_2_ and Co-VO_2_. When the energy of Co ions becomes 100 keV, structural distortion, particularly at V sites, is somewhat reduced, compared to that at low ion energy, as shown in Fig. 9(d). This is not observed in Cr-VO_2_. The positions and shapes of the EXAFS peaks from Cr-VO_2_ with a Cr ion flux of 5 × 10^13^ ions/cm^2^ are significantly different from those of other specimens, implying serious distortion existing in all atomic sites. Interestingly, the position and shape of the third two peaks near 4.0 Å in Fig. 9(c, d) are similar to those of the pristine VO_2_, although the intensity is quite weak. The third peaks mainly correspond to further V atomic shells beyond a conventional cell of a rutile-phased VO_2_. Those peaks of Cr-VO_2_ with a flux of 5 × 10^13^ ions/cm^2^ show a slight temperature-dependent behavior, as shown Fig. 8(c, d). This is a further evidence that the V sites of Cr-VO_3_ with a flux of 5 × 10^13^ ions/cm^2^ still experience a weak SPT during both heating and cooling processes.

## Discussion

Many researchers have observed that the MIT of a single crystal VO_2_ occurs simultaneously with its SPT at T_c_ ≈ 68 °C^5,29^. Since the MIT of VO_2_ is accompanied by an SPT, the contribution of each structural change, such as VO_6_ octahedrons, V–V dimers, and vertex V arrays, on the MIT is indistinguishable because the changes occur *simultaneously* in a single crystal VO_2_. EXAFS from Cr-VO_2_ and Co-VO_2_ films shows independent changes of structural properties of atomic shells during MIT. The direct comparison of electrical resistance and EXAFS measurements suggests that the contribution of the VO_6_ octahedrons on the MIT is negligible. The energy states of V 3d orbitals are split in the *e*_g_ and *t*_2g_ bands due to the crystal field effects of a VO_6_ octahedron in VO_2_. In Cr-VO_2_ with a flux ≥ 10^13^ ions/cm^2^ the V–O pairs are significantly disordered, so that the VO_6_ octahedrons cannot have regular splitting of the *e*_g_ and *t*_2g_ bands. This prevents any regular alignment of V 3d orbitals in the specimen and can exclude the possibility of conduction electrons jumping from a lower energy band of the $${d}_{xy}$$ and $${d}_{xz}$$ orbitals to a higher energy band of the $${d}_{\parallel }$$($${d}_{{x}^{2}-{y}^{2}})$$ to trigger the metallic phase VO_2_. A lack of temperature-dependent features of the pre-edge peak from Cr-VO_2_ with a flux ≥ 10^13^ ions/cm^2^ is further evidence of no regular splitting of V 3d states because the pre-edge peak corresponds to the *e*_g_ and *t*_2g_ bands^7^. A dramatic increase of the pre-edge peak intensity of Cr-VO_2_ indicates an increase of local density of states in the V 3d orbitals due to the structural distortion of VO_6_ octahedrons. The pre-edge peak intensity of the V K edge increased and decreased for V_2_O_5_ and V_2_O_3_, respectively, relative to that of VO_2_, because there are more empty states in the V 3d orbitals of V_2_O_5_ than of V_2_O_3_^41,55^.

The dull pre-edge peak of VO_2_ at the V K edge consists of two peaks which correspond to the V 3d *t*_2g_ and *e*_g_ states, respectively, with an energy gap of ~ 2.0 eV^7^. XANES cannot detect the direct band gap because of its resolution limit. XANES from pristine VO_2_ shows that the intensities of the *t*_2g_ (lower peak) and *e*_g_ (upper peak) states decreases and increases, respectively, with no change of the band gap between the two states during heating^7^. As a result, the pre-edge peak seems to be shifted towards a higher energy for heating, as shown in Fig. 4(b, d). The pre-edge peaks of Cr-VO_2_ with a flux ≥ 10^13^ ions/cm^2^ show that the *t*_2g_ band at ~ 5467.5 eV nearly disappears while the peak intensity of the *e*_g_ states at ~ 5469.5 eV is very strong with no temperature dependence, as shown in Fig. 5(b, d). For the Cr-VO_2_ films with a flux ≥ 10^13^ ions/cm^2^, the lack temperature dependence of the pre-edge peak strongly implies no changes in the local density of states of the V 3d orbitals in the temperature range of 30–100 °C, although the films experience MIT and SPT. This is evidence indicating that the *t*_2g_ and *e*_g_ bands split by the crystal field effects are irrelevant to the MIT of VO_2_ and that an SPT of short-range orderings around the V atoms in VO_2_ does not directly contribute to the MIT. EXAFS shows an SPT and no SPT at the O sites of Cr-VO_2_ with a flux of 10^13^ and 5 × 10^13^ ions/cm^2^, respectively, while an SPT is observed at the V sites for both fluxes. This implies that the pre-edge peak of VO_2_ is mainly contributed by the nearest neighboring O atoms, rather than by the second neighboring V atoms. This contrasts to the previous studies of the pre-edge peak of transition metals, in which the authors discussed the contribution of the second neighboring atoms on the pre-edge peaks^41,44,56^.

On the other hand, traditional band theory cannot predict the bandgap of ~ 0.65 eV for M1-phased VO_2_ at room temperature^6^. Based on a structural-driven Peierls transition mechanism, two different distances of V–V pairs in M1 VO_2_ were introduced to understand the insulating phase of VO_2_^6,27,57–60^. The dimerization model also cannot explain the measured bandgap of VO_2_ at room temperature^6^. Our EXAFS measurements and calculations [Supplementary Materials] on Cr-VO_2_ with a Cr flux of 5 × 10^13^ ions/cm^2^ reveal a substantial amount of structural disorder at both oxygen and vanadium sites. The linear defects created due to the implanted ions are placed perpendicular to the current direction in the DC electrical resistance measurements of the films. Cr-VO_2_ barely maintains a crystalline structure without regular V–V dimers and does not show an SPT of V–V dimers due to the enormous amount of structural disorder and distortion in the V sites, although there are obvious MITs during both heating and cooling. The distances of the V–V dimers are approximately ~ 2.5 Å and ~ 3.2 Å in the M1 phase and become ~ 2.8 Å in the rutile phase^27,36^. Our result is further evidence that a V–V dimerization model cannot explain the MIT mechanism of VO_2_. However, a structural-driven Peierls transition may not be excluded as an explanation for the MIT of VO_2_ because the EXAFS peaks which mainly correspond to the V sites show a weak SPT at T_c_, as shown in Fig. 8(c, d). The sharp MIT features of the resistance curve from Cr-VO_2_ with a Cr ion flux of 5 × 10^13^ ions/cm^2^ strongly suggest a transition of interaction between conduction electrons at T_c_. The EXAFS and resistance measurements of the Cr-VO_2_ film support that the MIT is highly related to the interaction of conduction electrons and is triggered by the alignment of the V atomic arrays near T_c_.

Previous studies reported that the T_c_ values of Cr-added and Co-added VO_2_ shifted towards higher and lower temperatures, respectively^37–40,42^. The T_c_ values of both Cr- and Co-VO_2_ films with a flux ≥ 5 × 10^13^ ions/cm^2^ shift towards a higher temperature. Since the concentration ratio of the implanted ions is only ~ 0.023% for an ion flux of 10^14^ ions/cm^2^, the doping effects of the ions could be negligible. V_1−x_Ti_x_O_2_ also showed that the T_c_ decreased and increased for low and high concentrations of Ti^4+^, respectively^44^. Ti^4+^ ions which are mostly replaced at the V^4+^ sites of VO_2_ can cause the disorder and distortion of the V sites without doping effects. Previous studies of heavy ion irradiation with high energy on VO_2_ showed that the resistivity and the T_c_ value of VO_2_ were considerably modified due to an extra structural disorder^61,62^. Hofsäss and coworkers showed that 1 GeV ^238^U swift heavy ions substantially decreased the T_c_ value of VO_2_, although no surface hillocks were observed^61^. When 200 meV Ag^9+^-ions with a high flux bombarded VO_2_, the surface and the crystal symmetry of VO_2_ were seriously damaged. Both T_c_ value and resistivity jump size of the MIT of VO_2_ continuously decreased when the fluence of Ag^9+^ ions increased^62^. This result is somewhat different from that of Cr-VO_2_, Co-VO_2_, and V_1−x_Ti_x_O_2_, as discussed above. Since most of implanted Cr and Co ions remain in VO_2_ films, they play as impurities in addition to the ion tracks. Impurities in VO_2_ can modify the band structure, contribute the charge carrier density of the conduction band, disturb the SPT, and interrupt the propagation of electrons. When Cr concentration increased in VO_2_, both lattice constants and structural disorder of V_1−x_Cr_x_O_2_ increased, while the T_c_ value moved towards a higher temperature during both heating and cooling^38,39^. This is comparable to the T_c_ behavior of Cr-VO_2_ with the ion flux of 5 × 10^13^ ions/cm^2^.

The resistance and EXAFS measurements of the Cr- and Co-VO_2_ films with different ion energies and fluxes show that the SPT always occurs before and after the MIT during heating and cooling, respectively. This indicates that a percolation effect is negligible in the systems and that an SPT, particularly the V atomic arrays, is an essential prerequisite for the MIT of VO_2_. This corresponds to that of the pristine VO_2_. A few defects in VO_2_ assist SPTs during heating and cooling whereas many defects interrupt SPTs. As a result, △T_c_ (T_c heating _− T_c cooling_) becomes small and large, as shown in Figs. 1 and 2, respectively. When the concentration of defects is larger than a critical value, the MIT of VO_2_ can be totally destoryed, as reported in previous studies^61,62^. The total amount of defects in a film due to implanted ions increases with increase in the flux and the penetration depth of the ions because the ions create linear tracks. When the energy of the implanted ions increases, the penetration depth is expanded, leading to the creation of more defects in the film. This scenario is consistent with the results of the Cr- and Co-VO_2_ films with different energies and the same flux. For an ion flux of 10^14^ ions/cm^2^, MIT features from Cr-VO_2_ are significantly reduced while an obvious MIT is observed from Co-VO_2_. EXAFS reveals that Cr ions more seriously affect the O sites of VO_2_ than those of Co ions, as shown in Figs. 8 and 9. The penetration depths of the two ions on VO_2_ are roughly the same, as shown in the supplementary materials. Researchers observed that the T_c_ values of V_1−x_Cr_x_O_2_ and V_1−x_Co_x_VO_2_ increased and decreased, respectively, relative to that of pristine VO_2_^38,39,42^, This implies that the contributions of Cr and Co ions on the MIT of VO_2_ are not the same, as they interrupt and assist the SPT, respectively. EXAFS measurements reveal that Cr ions more effectively destroy the crystalline structure of VO_2_ than Co ions do. The different effects of Cr and Co ions to VO_2_ could be attributed to the different radii and the different oxidation states of Cr^3+^ and Co^2+^ ions. This study indicates that the T_c_ of VO_2_ can be increased or decreased by careful selection of a proper species of ions with different energies and fluxes.

## Conclusions

For the Cr and Co ion fluxes ≤ 10^14^ ions/cm^2^, both Cr- and Co-VO_2_ show sharp MIT features near T_c_. The T_c_ of both the Cr- and Co-VO_2_ films with a low ion flux is lower than that before ion implantation, while it shifts toward a higher temperature for a high ion flux. This indicates that the T_c_ of VO_2_ can be engineered by properly selecting the flux, energy, and species of ion beam. Both Cr and Co ions create a substantial amount of structural disorder and distortion in VO_2_. Based on resistance and EXAFS measurements, model calculations suggest that a sharp and abrupt MIT and SPT can occur in VO_2_ unless more than 5% of the V sites are disturbed by impurities. Temperature-dependent XANES from Cr-VO_2_ at the V K edge showed that the pre-edge peak alone cannot fully describe either the MIT or the SPT. Temperature-dependent resistance and EXAFS measurements reveal that crystal field splitting in the VO_6_ octahedron of VO_2_ does not play a critical role in the MIT. These study results suggest that an SPT of the V atomic arrays and the interaction of V 3d^1^ electrons are the necessary conditions for the MIT of VO_2_, supporting both the structural-driven-Peierls and Mott–Hubbard models. This study also shows that ion-implantation techniques can be widely used to engineer the T_c_ and the MIT of VO_2_, and particularly of VO_2_ nanostructures, without degrading the sharpness of the MIT features^23^.

## Methods

### Synthesis of VO_2_ films

The *b*-oriented VO_2_ films were fabricated on α-Al_2_O_3_(0001) substrates using direct current (DC)-sputtering deposition from a vanadium target with a purity of 99.95%. The base vacuum of the growth chamber was 10^–6^ Torr and the pressure was kept at 10^–3^ Torr during the deposition. Ar gas was used as the plasma and the substrate temperature was maintained at ~ 500 °C. After deposition, the films were annealed at 500 °C for 30 min with a mixture gas flow of Ar: O_2_ = 300:1. More details of VO_2_ film fabrication can be found elsewhere in the literatures^7,36^.

### Cr and Co ion implantation on VO_2_ films

Cr and Co ions with an energy of 30–100 keV and a flux of 10^12^–10^14^ ions/cm^2^ vertically bombarded the VO_2_ films at room temperature in a vacuum at the Korean Multi-Purpose Accelerator Complex (KOMAC).

### In-situ XAFS measurements

Temperature-dependent XAFS measurements were conducted from Cr-VO_2_ and Co-VO_2_ films and a pristine VO_2_ film as a counterpart at the V K edge (5465 eV). XAFS measurements were performed with a fluorescence mode using a Si(111) double-crystal monochromator at beamline 8C of the Pohang Light Source (PLS) and beamline 20-BM of the Advanced Photon Source (APS). The XAFS data were taken with an unpolarized geometry where the angle between the film surface and the incident X-ray beam was fixed at 45 degrees. During *in-situ* temperature-dependent XAFS measurements, the DC electrical resistance was *simultaneously* measured from the same specimens^7,33^. A thermocouple was directly contacted to the surface of a VO_2_ film to accurately measure the true temperature of the film in real time. The resistance and the temperature were recorded after the temperature at each set temperature was stabilized. The temperature was monitored and controlled within ± 0.1 degree during the XAFS scans and the resistance measurements. Each XAFS scan took approximately 15 min.

### DC resistance measurements

Two-probe DC-resistance measurements were performed from pristine, Cr-VO_2_, and Co-VO_2_ films before ion-implanted at the applied voltage of 0.5 V using a Keithley 2400 SourceMeter^7,36^. After the ions were implanted, the resistance measurements were simultaneously performed with in-situ XAFS measurements.

## Supplementary information

Supplementary Information
