# Supplementary material for: Decoupling the metal insulator transition and crystal field effects of VO2
Source: Sci Rep. 2021 Feb 4;11:3135. doi: 10.1038/s41598-021-82588-4 (PMC7862372; doi:10.1038/s41598-021-82588-4)
Supplement: Supplementary file 1 — Supplementary Information [file 41598_2021_82588_MOESM1_ESM.docx]

**Supplementary Materials**

**Decoupling the Metal Insulator Transition and Crystal Field Effects of VO_2_**

In-Hui Hwang, Chang-In Park, Sunmog Yeo, Cheng-Jun Sun, and Sang-Wook Han^*^

^*^Correspondence and requests for materials should be addressed to S.-W.H. (shan@jbnu.ac.kr).

**1. Temperature-dependent XAFS from VO_2_ films**

Figures S1, S2, and S3 show raw EXAFS data in *k*-space from pristine VO_2_, Cr-VO_2_, and Co-VO_2_ films. The EXAFS data correspond to Figs. 7, 8, and 9, respectively, before Fourier transformed into the *r*-space. EXAFS data in the *k*-space was directly extracted from raw XAFS data in Figs. 7, 8, and 9 measured as a function of the incident x-ray energy using the IFEFFIT software package [53]. The photoelectron wave number, k, is determined as $k=\sqrt{2m_{e}(E-E_{0})}/\hbar$, where *m*_e_ is the electron mass, *E* is the incident x-ray energy, and *E*_0_ is the absorption edge energy. For the Fourier transform of EXAFS data, the Hanning window with the windowsill width of 1.0 Å^-1^ were used. EXAFS was analyzed along a standard EXAFS data analysis procedure [54]. EXAFS data in the *k*-space from Cr-VO_2_ and Co-VO_2_ films shows substantial changes, compared to that of the pristine VO_2_, as shown in Figs. S1, S2, and S3. The structural changes can be more obviously seen in the *r*-space than in the *k*-space. EXAFS data in the k range of 2.5 – 10.5 Å^-1^ were used in the Fourier transform into the *r*-space, as shown in Figs. 7, 8, and 9 in the main text.


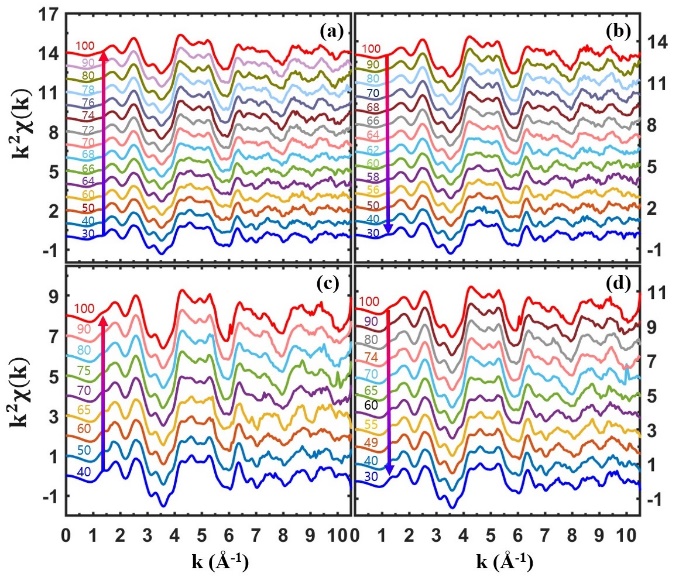


Figure S1. (a) and (b) EXAFS (k^2^χ) from the pristine VO_2_ film during heating and cooling processes, respectively, as a function of the photoelectron wave number, k. (c) and (d) EXAFS from the Cr-VO_2_ film for the Cr ion energy of 50 keV and the flux of 10^12^ ions/cm^2^ during heating and cooling, respectively. The data corresponds to the Fourier transformed EXAFS data in Fig. 7 in the main text.


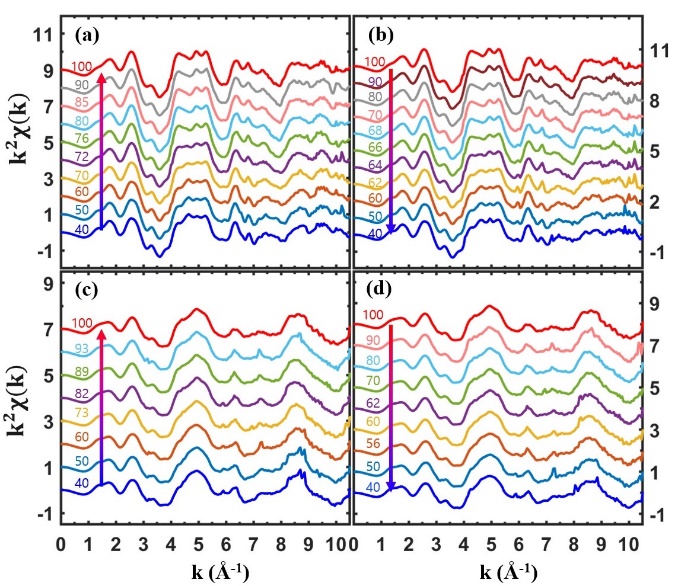


Figure S2. (a) and (b) EXAFS (k^2^χ) from the Cr-VO_2_ film for the Cr ion energy of 50 keV and the flux of 10^13^ ions/cm^2^ during heating and cooling processes, respectively, as a function of the photoelectron wave number, k. (c) and (d) EXAFS from the Cr-VO_2_ film for the Cr ion energy of 50 keV and the flux of 5ⅹ10^13^ ions/cm^2^ during heating and cooling, respectively. The data corresponds to the Fourier transformed EXAFS data in Fig. 8 in the main text.


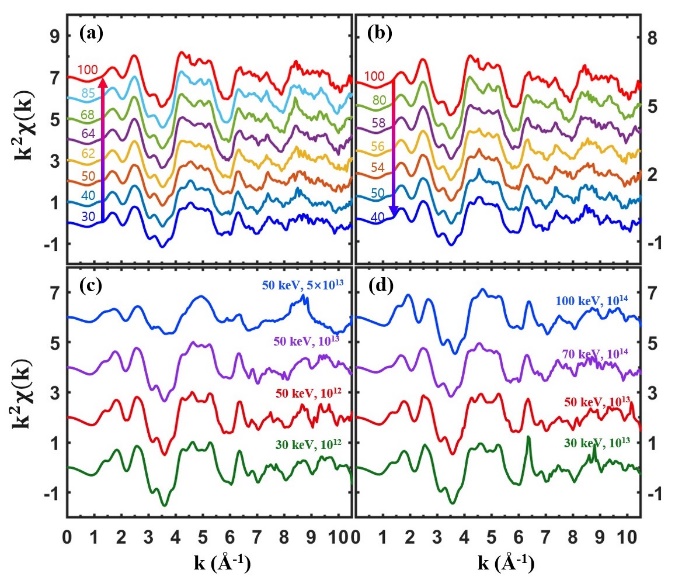


Figure S3. (a) and (b) EXAFS (k^2^χ) from the Co-VO_2_ film for the Co ion energy of 70 keV and the flux of 10^14^ ions/cm^2^ during heating and cooling processes, respectively, as a function of the photoelectron wave number, k. (c) and (d) EXAFS from the Cr-VO_2_ and Co-VO_2_ films in the insulating phase, respectively, for different ion energies and fluxes. The data corresponds to the Fourier transformed EXAFS data in Fig. 9 in the main text.

**2. Distribution of Implanted Cr and Co Ions in VO_2_**

Figure S4 presents the longitudinal distribution of Cr ions with different energies in VO_2_ estimated by using the SRIM software package [45]. The mean penetration depth of Cr ion with an energy of 30 keV, 50 keV, 70 keV, and 100 keV is 181 Å, 280 Å, 380 Å, and 515 Å, respectively. The population of the implanted ions as a function of distance from the film surface follows a near Gaussian function. The full width at half maximum (FWHM) of the Cr ion distribution in VO_2_ is 74 Å, 112 Å, 145 Å, and 191 Å for a Cr ion energy of 30 keV, 50 keV, 70 keV, and 100 keV, respectively. The penetration depth of Co ions is similar to that of Cr ions with the same energy. Figure S5 shows the Co ion distribution in VO_2_ for a Co ion energy of (a) 30 keV, (b) 50 keV, (c) 70 keV, and (d) 100 keV. When the energy of implanted Co ions increases from 30 keV to 100 keV, the penetration depth of the ions changes from 169 Å to 480 Å and the FWHM increases from 68 Å to 179 Å, respectively, as shown in Fig. S5.


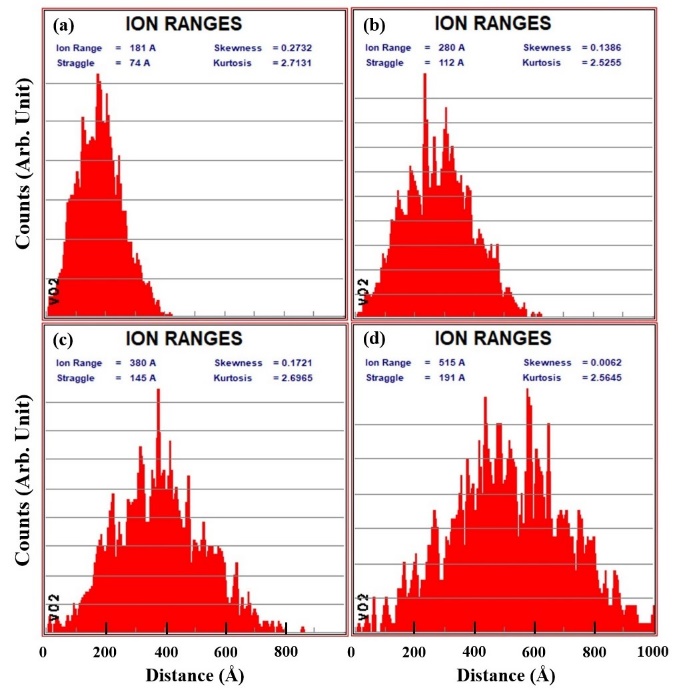


Figure S4. The calculated distribution of Cr ions with different energies, (a) 30 keV, (b) 50 keV, (c) 70 keV, and (d) 100 keV, respectively, in VO_2_ as a function of distance from the surface.


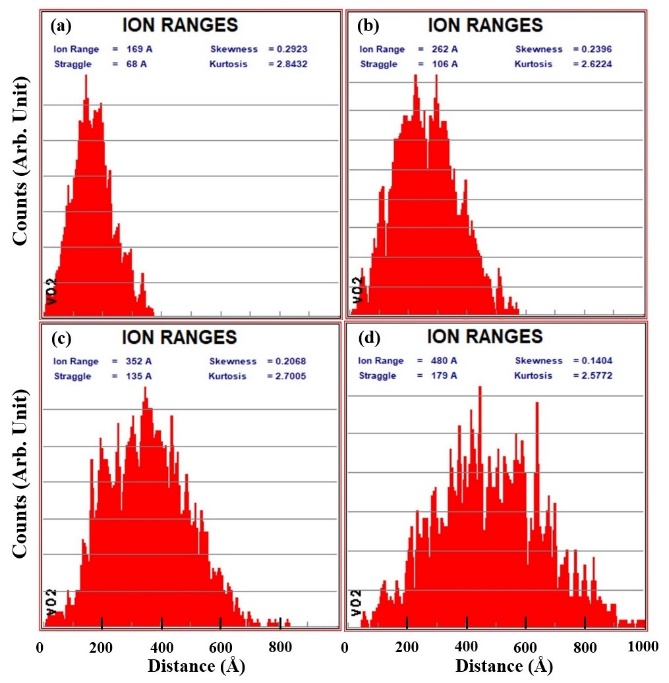


Figure S5. The calculated distribution of Co ions with different energies: (a) 30 keV, (b) 50 keV, (c) 70 keV, and (d) 100 keV, in VO_2_ as a function of distance from the surface.

The maximum distance reached by the implanted ions in a material can be estimated using the mean penetration depth and the FWHM of the distribution function of implanted ions. The practical maximum distance reached by implanted ions in a film can be reasonably estimated by the summation of the mean penetration depth and approximately three times the longitudinal FWHM. For the case of Cr ions with an energy of 50 keV, the maximum distance can be ~612 Å, which is similar to the estimated distance, as shown in Fig. S4 (b). The thickness and the mean grain size of the VO_2_ film are ~130 nm and ~170 nm, respectively [36]. When a VO_2_ film consists of grains, implanted ions can reach the bottom of the film through the boundaries of the grains because the electron density of the grain boundaries can be lower than that of the middle parts of the grains. Furthermore, ions can be implanted through the lateral surfaces of the grains because the incident ion beam has an angular dispersion. Ion-implantation on lateral surfaces has been observed on vertically aligned ZnO nanorods [46]. This scenario can explain the insulator characteristics of the Cr-VO_2_ film with a Cr energy of 50 keV and a flux of 10^16^ ions/cm^2^ in the temperature range of 30 – 100^o^C, as shown in Fig. S6.


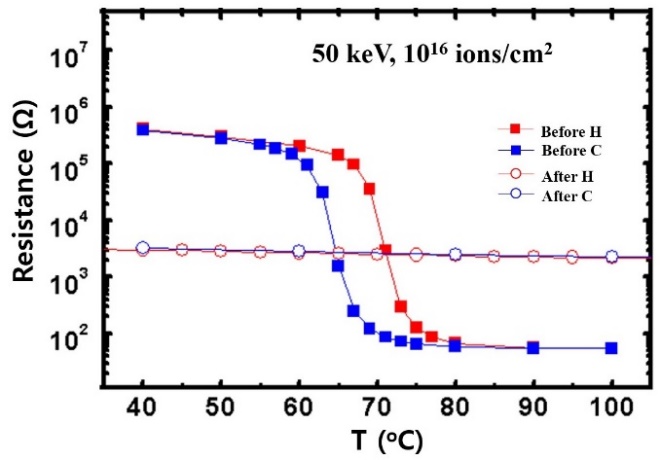


Figure S6. Temperature-dependent resistance from the Cr-VO_2_ film with an energy of 50 keV and a flux of 10^16^ ions/cm^2^ before and after Cr ion implantation during heating and cooling.

Before Cr ions with a flux of 10^16^ ions/cm^2^ were implanted, the electrical resistance curves of the film showed sharp transitions at 72^o^C and 65^o^C during heating and cooling, respectively. The MIT features of the film completely disappeared after the ions were implanted. The results of the resistance and XAFS measurements strongly suggest that the entire VO_2_ film is affected by the implanted Cr and Co ions with the ion energy of 30 – 100 keV. For the low flux of Cr and Co ions, the distribution of the implanted ions may not be uniform in the VO_2_ films, although the ions reach the bottom of the film. The parts near the surface and the grain boundaries of VO_2_ film can have more structural disorder and distortion than the middle-bottom parts of the grains. The thermal energy which induces an SPT is relatively lower in a VO_2_ cell with a small amount of structural disorder than a perfect VO_2_ cell because a slight disorder in atomic distances can make VO_2_ somewhat easily changed between M1 and rutile phases. EXAFS showed that the distance and the disorder of V-V pairs of the Cr-VO_2_ film with 10^12^ ions/cm^2^ were not changed much, as shown in Fig. 9 (c), although the T_c_ values and the FWHM of the resistance loop was significantly changed relative to those of VO_2_ before ion implantation, as shown in Figs. 1 (a) and (b). This indicates that the MIT of VO_2_ is mainly affected by an SPT of the V-V arrays.

SRIM calculations show that most of the implanted ions with a lower energy are distributed near the surface. Since the VO_2_ film consists of grains with a mean size of ~170 nm and the ion beam has an angular deviation, the implanted ions can affect the entire film through the grain boundaries and the lateral surfaces of the grains. Since the x-ray penetration depth on VO_2_ at the V K edge is ~6.5 μm, which is much longer than the film thickness of 130 nm, the x-rays detect the mean structural properties of the entire film.


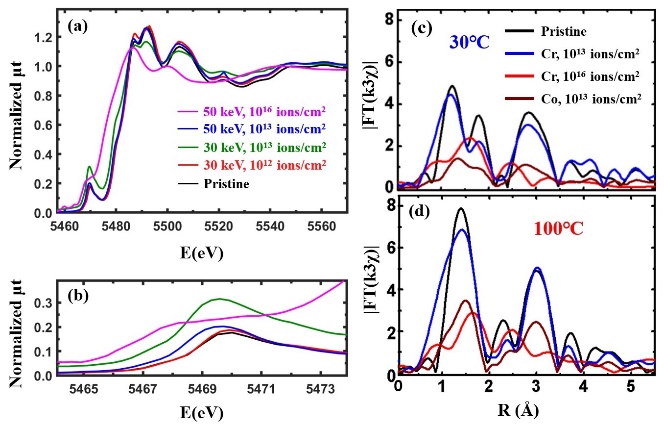


Figure S7. (a) Normalized total x-ray absorption from Cr-VO_2_ and pristine VO_2_ films at the V K edge as a function of the incident x-ray energy at 30^o^C and (b) the expansion of the pre-edge peak shown in (a). (c) and (d) Magnitude of Fourier transformed EXAFS from the Cr-VO_2_ and pristine VO_2_ films as a function of distance from a V atom at 30^o^C and 100^o^C, respectively.

Figure S7 shows x-ray absorption near edge structure (XANES) from Cr-VO_2_ with various energies and fluxes at the V K edge. XANES from the Cr-VO_2_ film with the energy of 50 keV and the flux of 10^16^ ions/cm^2^ shows that the main absorption edge shifts by 5 eV toward to a lower energy and the pre-edge peak becomes dull, relative to those of the pristine VO_2_. The changes of XANES from the Cr-VO_2_ film strongly suggest that the VO_2_ film does not remain in a wurtzite phase. This indicates that the Cr ions with the energy of 50 keV affect entire VO_2_ cells in the film and does not agree with the SRIM calculations of a single VO_2_ crystal. XAFS measurements correspond to the scenario of implanted ions distributed over the entire VO_2_ film through the grain boundaries and the lateral surfaces of grains. The local structural properties around the V atoms can be more obviously seen by extended XAFS (EXAFS), which shows small oscillations above the absorption edge.

Figure S7 (c) and (d) show the Fourier transformed EXAFS of the Cr-VO_2_ films with various energy and fluxes at 30^o^C and 100^o^C, respectively. A pristine VO_2_ film shows different local structures at 30^o^C and 100^o^C, implying M1 and rutile (or M2) phases, respectively, whereas the Cr-VO_2_ film with a flux of 10^16^ ions/cm^2^ has a same structure without an SPT in the temperature range of 30^o^C – 100^o^C. The structure of Cr-VO_2_ with a flux of 10^16^ ions/cm^2^ is very different from that of the pristine VO_2_, which indicates that the entire Cr-VO_2_ film does not have either an M1 phase at room temperature or a rutile (or M2) phase at 100^o^C. This corresponds well to the resistance measurements of the Cr-VO_2_ films. The implanted Cr and Co ions may not uniformly distribute over the Cr- and Co-VO_2_ films. However, the pre-edge peak and EXAFS indicate that most of VO_2_ cells in the films are affected by the Cr and Co ions with an energy range of 30 – 100 keV. The resistance and XAFS measurements on Cr-and Co-VO_2_ films show that the SPT occurs prior to the MIT during heating. This indicates no percolation effects on the films [36]. A lack of percolation effects is an evidence that the distribution of Cr and Co ions on Cr- and Co-VO_2_ films does not decisively influence the MIT when most of VO_2_ cells are affected by the implanted ions.

For a Cr ion flux of 10^13^ ions/cm^2^, the concentration of Cr ions in a film with a surface area of 1×1 cm^2^ and thickness of 1300 Å can be estimated to be 7.7×10^17^ ions/cm^3^. The concentration of vanadium atoms in VO_2_ is approximately 3.3×10^22^ atoms/cm^3^. Cr ions can be partially substituted into the V sites and become interstitially placed. If most Cr ions are randomly substituted into the V sites of VO_2_, the ratio of V sites substituted by Cr ions is roughly 0.0023% for a Cr-ion flux of 10^13^ ions/cm^2^. Similarly, the substitution ratio of V sites by Cr ions for a Cr-ion flux of 10^16^ ions/cm^2^ is estimated to be 2.3%. Cr and Co ions with the concentration of 0.0023% is undetectable by XAFS due to its resolution limit. XAFS measured from the Cr-VO_2_ films with the ion concentration of even 2.3% does not provide useful information of the local structural and the chemical properties of the ions.

Implanted ions more significantly affect the structural properties of a parent system due to their tracks [46] than do any impurities that are added during growth. If a *b*-oriented VO_2_ film is uniformly bombard with Cr ions with a flux of 10^13^ ions/cm^2^, the Cr ions will hit approximately 2.26 % of the cells on the film surface. When an ion vertically bombards the surface of a film with a thickness (or the maximum distance of ions reaching) of *L*, the probability of a cell in [0,*L*] being hit by the ion is described by the following equation:

$P\left( x \right)=1-A\int_{0}^{x} F\left( x \right)dx$ (1)

where *P*(*x*) is the probability of a cell being hit by the ion at a distance, *x*, from the surface, *A* is the normalization factor, and *F*(*x*) is the distribution function of an implanted ion. The ion distribution may follow a Gaussian function, as

$F\left( x \right)= e^{-\alpha{(x-l_{p})}^{2}}-y(x)$ (2)

where *y*(*x*) is a linear function which satisfies the boundary condition of *F*(0) = *F*(*L*) = 0. Figure S8 shows the probability, *P*(*x*), under the assumption of a Gaussian distribution of implanted ions. The probability shows that cells near the surface can be highly affected by the implanted ions. The mean probability of a cell in [0,*L*] being hit by the ion can be estimated by using the average probability, as

$\bar{P}= \frac{1}{L}\int_{0}^{L} P\left( x \right)dx$ (3)

where *P*(*x*) is the probability function in Eq. (1).


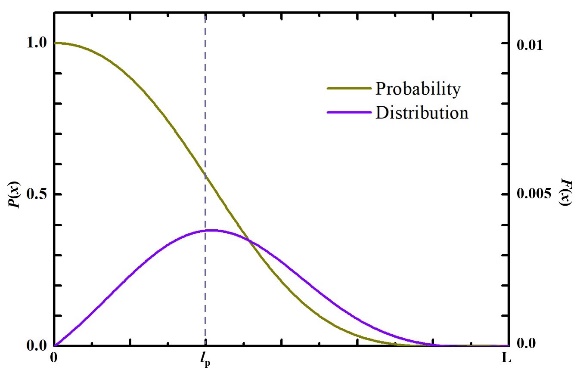


Figure S8. The probability, *P*(*x*), of an atomic cell in [0,*L*] being hit by an implanted ion with a penetration depth of *l*_p_ and a Gaussian distribution, *F*(*x*), as function of distance, *x*, from the surface.

The mean probability of a cell in a VO_2_ film with a thickness of 1300 Å being hit by a Cr ion with an energy of 50 keV is estimated to be ~0.22, assuming *l*_p_ = 280 Å and a Gaussian distribution with FWHM = 180 Å. For the Cr ions with an energy of 50 keV and a flux of 10^13^ ions/cm^2^, the probability of a cell in the film with a thickness of 1300 Å being hit by a Cr ion is estimated to be ~2.26% × 0.22 ≈ 0.5%. When the flux of Cr ions is 10^16^ ions/cm^2^, a VO_2_ cell in the film will be hit by approximately five Cr ions, which can seriously damage the cells of the VO_2_ film and thereby disrupt the original crystalline symmetry. Particularly, cells near the surface can be totally destroyed.
